# Engaging Native American High School Students in Public Health Career Preparation Through the Indigenous Summer Enhancement Program

**DOI:** 10.3389/fpubh.2022.789994

**Published:** 2022-02-22

**Authors:** Heather M. Dreifuss, Kalvina L. Belin, Jamie Wilson, Shawndeena George, Amber-Rose Waters, Carmella B. Kahn, Mark C. Bauer, Nicolette I. Teufel-Shone

**Affiliations:** ^1^Diné College, Shiprock, NM, United States; ^2^Center for Health Equity and Research, Northern Arizona University, Flagstaff, AZ, United States

**Keywords:** Native American, Indigenous health, Indigenous framework, Navajo, high school, STEM, peer mentor, American Indian

## Abstract

Native American^1^ populations are systematically marginalized in the healthcare and public health workforce. One effective approach to reduce health disparities and improve health care delivery among Indigenous populations is to train more Native American health professionals who integrate academic and cultural knowledge to understand and influence health behaviors and perspectives. Diné College partnered with Northern Arizona University to develop the Navajo Native American Research Center for Health (NARCH) Partnership, funded by the National Institutes of Health. The high school component of the Navajo NARCH Partnership created the Indigenous Summer Enhancement Program (ISEP), a 1-week summer training program providing exposure to health careers and mentorship in pursuing public health careers for Native American high school students. ISEP utilizes the Diné Educational Philosophy (DEP), a Navajo conceptual framework to serve as the foundation of the program. In 2020–2021, due to COVID-19 restrictions, the DEP model had to be incorporated in the Navajo NARCH high school virtual program activities. ISEP used 2018 and 2019 past program evaluation data to inform the virtual programming. Students' perception of the program was collected using an online Qualtrics evaluation questionnaire. Students stated appreciation for program staff, fellow students, peer mentors and culturally relevant learning experiences in both virtual and in-person environments. Recommendations included: expanding the length of ISEP and continuing the hands-on activities and Public Health Leadership series.

## Introduction

Native American (NA) populations are systematically marginalized in the healthcare and public health workforce. Native American students have the lowest percentage of college enrollment; 19.6% earn at least a bachelor's degree when compared to 35.8% of non-Hispanic whites (1). In 2019, NA high school students were less likely to pursue higher education due to the highest dropout rate at 9.6% compared to Whites at 4.1%, Blacks at 5.6%, and Hispanics with 7.7% (2). According to the American Council of Education, 70.6% of NA students enrolled in bachelor's degree seeking programs attend a public 4-year institution, where 6.8% pursued a major in a public health or health sciences field. Not only are NA students disproportionately underrepresented in higher education, but they are also disproportionately underrepresented in the public health and health sciences fields (3).

Barriers that NA students face to complete a post-secondary education includes health disparities, discrimination in health care delivery, cultural differences, low socioeconomic status, historical trauma, and underrepresentation in the health care professional workforce (4–6). In the 2010 Census, 22% of American Indian and Alaska Natives (AI/AN) that live on reservations or other trust lands were living in poverty in comparison to non-Hispanic whites at 9.6% (1). One effective approach to reduce health disparities and improve health care delivery among Indigenous populations is to train Native American health professionals who understand cultural influences on health behaviors and perspectives (5, 7–9).

On the Navajo Nation and in other tribal communities, sustainable Science, Technology, Engineering, and Math (STEM) pathway programs are needed at the high school and college levels; incentives to increase student engagement could include paid internships, dual credit programs, and service learning experiences. These pathway programs help guide and encourage students to pursue an education and career in a STEM-related area. For Diné (Navajo) people, providing culturally relevant pathway programs for high school students can stimulate their entry into health and public health professions that support the development and use of culturally and contextually concordant public health strategies, which in turn may increase the likelihood of their use in local communities (10). Through training and cultivating an understanding of their own cultural and strength-based assets, the future Diné public health workforce can be equipped to create effective methods to address health disparities in their communities (5, 7–9).

### Community Partnership

Recognizing the need for public health pathway programs, Diné College, a Navajo Nation tribal college, partnered with Northern Arizona University (NAU) to develop the Navajo Native American Research Center for Health (NARCH) Partnership funded by the National Institutes of Health's National Institute of General Medical Sciences (5S06GM123550). The Navajo NARCH Partnership aims to develop Diné scientists and health professionals to enhance research skills and knowledge among high school, undergraduate, graduate students, and Diné health department employees. The Navajo NARCH Partnership's existing programs include a NAU Masters in Public Health with an Indigenous Health track, a Diné College 4 year undergraduate degree program in public health, a 10-week Summer Research Enhancement Program (SREP) for Indigenous undergraduate students (11) and high school outreach programs. During the academic year, current high school programs include dual credit classes through Diné College, monthly public health seminars, internships with local public health programs, and a service-learning program in collaboration with the Navajo Epidemiology Center and Diné College public health programs. The high school component of the Navajo NARCH Partnership, in collaboration with Central Consolidated School District's Shiprock High School located on the eastern side of the Navajo Nation, created the Indigenous Summer Enhancement Program (ISEP), a 1 week summer training program that provides exposure to and mentorship in pursuing public health careers for Native American high school students.

## Pedagogical Framework

Inspired by the successful application of the Diné traditional living system, known as the Diné Education Philosophy (DEP), for the foundation of undergraduate courses and the public health SREP for Native American undergraduate students (12), ISEP also is guided by the culturally grounded DEP model. The DEP model, created by Diné cultural specialists at Diné College, is based on the four phases of the Diné way of life (13). The first phase is *Nitsáhákees* (Thinking), the second phase is *Nahat'á* (Planning), the third phase is *Iiná* (Living) and the fourth phase is *Sihasin* (Assuring). Each phase represents cultural elements and values associated with the natural processes of life, such as daily living activities. This article describes the innovative strategies used to adapt the DEP model for high school students as well as the application of peer mentoring in the ISEP program. This article presents qualitative and quantitative outcomes from program evaluations conducted after the implementation of ISEP and highlights the cultural relevance and acceptability of the public health summer program. In addition, this article will explore both the original innovation and the incorporated changes after the program moved to a virtual format.

## Learning Environment

### Program Description: Indigenous Summer Enhancement Program

ISEP is a 1-week program that promotes the field of public health, health research, and health professions among high school students, grades 9th through 12th. This program is distinctive in its incorporation of Diné cultural values and models. The overall learning objectives for ISEP include (1) increase knowledge of public health careers and research opportunities on the Navajo Nation; (2) identify with successful Native American public health professionals and college students, (3) apply cultural models to career pathway planning, and (4) learn and apply digital storytelling skills to develop a culminating project. The ISEP curriculum begins by introducing the DEP model to high school students through Navajo traditional stories (14). Students learn these teachings by illustrating essential elements of the DEP model, and gaining knowledge of the associated colors, animals, mountains and sacred stones of each direction. Then the DEP model is adapted for use with high school students by applying it to the college application process and career planning. ISEP introduces an indigenous public health lens that explores the Diné concept of *Hózhó*, “—beauty, balance, peace, wellness, and harmony” (15). Students apply the concept of *Hózhó* as a health promotion intervention activity when they reflect on *Hózhó* (protective) and risk factors in their own lives. The ISEP team introduces the *Hózhó* Resilience model, consisting of three main elements, (1) harmony in relationships, (2) respect, and (3) spirituality as an existing asset that can be used to design public health prevention strategies (16). The program opens with a Navajo prayer and introductions by clan, which is typical of Diné people to acknowledge existing relationships through kinship or common clans. This sets the stage for interactions among students and staff. The program closes with a talking circle, an in-depth reflection of the ISEP program's influence on students' future career directions and a Blessing Way and Protection Way Prayer to promote positive gains and guards against threats to health and wellbeing. These elements ground the ISEP program in Diné culture.

#### Peer Mentor Process

Mentorship strategies implemented into pathway programs have been shown to increase peer mentor's confidence, leadership abilities, and awareness about the importance of cultural diversity and community engagement (17, 18). An innovative peer mentor strategy, where youth serve as role models for other high school students, was implemented after students from the first ISEP cohort in 2018 requested to return and be part of ISEP again. As a result of the initial success, past ISEP participants or alums currently are recruited to serve as peer mentors for the subsequent year.

Prior to the first day of ISEP, peer mentors attend a training that includes a leadership self-assessment, communication tips and ways to operationalize their leadership skills. The leadership self-assessment, based on six key dimensions for mentors (19), invites peer mentors to reflect on their current areas of strength and growth. Both the self-assessment and skills for effective mentors were adapted from the LEAD Peer Mentor Program (19). First peer mentors reflect on the following questions: What helps you to be a good communicator? What do you need from others to communicate well? What does good communication look like? What does good communication sound like? When interacting with other students, how can you be supportive in your words and in your actions? Think of a time you felt supported, what does it look like/sound like to be supported? Then basic communication tips, such as being present, active listening, asking facilitative questions, using I statements, use of tone and paraphrasing to clarify and summarize are enforced through a series of situational role plays between peer mentors. Peer mentors prepare to co-teach lessons and assist with ISEP activities such as morning check-in, daily reflections, icebreakers and mentoring peers in digital storytelling.

#### Digital Storytelling Workshop

Digital stories are typically 3–5 min in length and are created in a collaborative workshop model. The digital storytelling (DST) process lends itself to strengthening social support systems by listening to others, being heard, and co-constructing collective meaning (20, 21). The DST workshop within ISEP serves as both a learning tool and a final assessment to synthesize the collective learning about public health career pathways and the planning required to pursue each of the core public health disciplines. Students work in DST teams of three to four students and use guiding questions to research the academic requirements for various public health careers, colleges and universities that offer these academic programs, estimated salary ranges, and potential jobs on the Navajo Nation. Students are encouraged to interview a Native public health professional to gain more understanding of how a specific public health discipline, such as epidemiology or child and family health plays an important role in their community and what a typical work day includes. This method is used to engage students to explore the different disciplines of public health and to reflect on their personal resilience or how their communities demonstrate resilience.

#### Implementation of Programmatic Changes

Due to unprecedented challenges presented by COVID-19, ISEP transitioned to an online format for the summers of 2020 and 2021. Kahn et al. (11) describes the adaptation of the SREP and ISEP programs. In 2020, a virtual model mirroring an online college course was implemented. The ISEP team used past evaluation data from 2018 to 2019 to guide the virtual programming. The content moved from 8 h days to two shorter blocks of time. New content was covered in the morning block and in the afternoon, students participated in a digital storytelling workshop, extending structured time dedicated to digital stories.

Based on the request for additional public health speakers and culturally related topics in the 2019 ISEP evaluation, in the 2020 virtual edition Indigenous health professionals were invited to participate in a Leaders in Public Health series. Each professional shared their personal career paths and presented on public health topics such as COVID-19, genetics, maternal and child health, and One Health. In 2021, the Leaders in Public Health series, purposively invited public health leaders actively working with projects on the Navajo Nation. These series focused on the leader's background, support systems and challenges on their academic pathway, current projects in public health on the Navajo Nation, project strategies to arrest COVID-19 infection, and specific leadership qualities used in their work. The ISEP team facilitated first hand access to local role models during the series by building in time at the end of the presentation for students to talk informally with these current Diné public health leaders.

Based on the suggestions from the ISEP 2018 and 2019 cohorts for more hands-on problem solving activities about public health and culturally related topics, the ISEP team implemented culturally grounded daily mindfulness and the Mini DEP Experiential Research Project, which spiraled and reinforced the DEP framework throughout the ISEP curriculum. Through mini-lessons on the DEP model, research process, and data collection and analysis basics, students experienced a mini research project from start to finish. The ISEP staff divided students into four research teams, each team consisted of three to four students, a peer mentor and an ISEP staff member. First students learned about the DEP model by identifying the four phases and their associated values & symbolism. Second, students learned about the research process to understand ethical research, institutional review boards, and how to formulate a research question. Next students engaged in the research process by applying the DEP model to their research protocol: (1) **Nitsáhákees:** Thinking: Develop a research question, (2) **Nahat'á:** Planning: Methods, (3) **Iiná:** Living: Data collection and analysis, and (4) **Sihasin:** Assurance: Draw conclusions and plan for dissemination (Table 1). As part of a mini lesson on data, students classified the difference between quantitative and qualitative data and learned how data can validate research questions. Students applied this to their team's project by identifying the data collection method and creating their team's data collection tool. Depending on the research question, students selected their data collection tool (e.g., questionnaires, photographs, or interviews). Then to embody **Iiná**, the ISEP students assumed the role of the researchers and the peer mentors assumed the role of the research participants, while students collected data from all of the peer mentors. Finally, students regrouped as research teams to analyze the data and prepare for a brief internal sharing of each mini research team's conclusions using a staff-created template in Google Slides.

**Table 1:** DEP research process and mini DEP experiential research activities.

**DEP term**	**Meaning**	**Research process**	**Activities**
*Nitsáhákees*	Thinking	Identify a research question	Develop a research question
*Nahat'á*	Planning	Select study approach Design study	Identify and create a data collection tool
*Iiná*	Living	Collect and analyze data	Collect and analyze data in research teams
*Sihasin*	Assurance	Draw conclusions Disseminate findings	Share findings internally with staff and peers using google slides template

## Results

### Data Collection and Data Analysis

A pre-/post-test design was conducted to evaluate the program and inform the next iteration of ISEP. Data was collected by an online Qualtrics program evaluation questionnaire (Table 2) survey. Students completed an evaluation questionnaire at baseline and at the conclusion of the program. They responded to the cultural relevance and understandability of the ISEP content, interest in college preparation, baseline and post public health knowledge, academic challenges and barriers, their further career aspirations, education, and suggested program improvements. A NARCH team member who did not mentor or instruct any student assignments administered the evaluation. Descriptive statistics were used to report the aggregate data using Excel. The open-ended responses were analyzed using a thematic analysis method.

**Table 2:** Program evaluation questions from post-survey with high school students.

Closed-ended questions based on Likert scale (strongly agree, agree, disagree, strongly disagree):
• This program provided me basic hands-on experience in health-related research • During the program, I was able to learn about ways I can advocate for the health of my community • During the program, I was able to learn about and consider various careers in the health field • This week-long experience of the Indigenous Summer Enhancement Program (ISEP) met my expectations
Open-ended questions:
• What did you like best about the program? • What did you like least about the program? • What suggestions would you make to improve this program in the future?

### ISEP Evaluation Data

The 2018–2021 ISEP cohort demographics (Table 3) remained similar even after the transition to a virtual format. The 2018 ISEP cohort consisted of eleven students, 73% identified as female. All eleven students self-identified as Native; ten as Diné and one as White Mountain Apache. The 2019 ISEP cohort included sixteen students, 75% identified as female. A majority (94%) of students self-identified as Native; fifteen as Diné and one as non-Native. The virtual 2020 ISEP cohort had ten students, 80% identified as female and all ten students self-identified as Native and Diné. The virtual 2021 ISEP cohort consisted of seventeen students, 82% identified as female. All seventeen students self-identified as Native; fifteen as Diné. Students' ages ranged from 14 to 18 years old in all four of the cohorts.

**Table 3:** ISEP cohort demographics.

	**Post-** **evaluation** **participants** **(***n***)**	**Female** **% (***n***)**	**Age** **(years old)**	**Self-identified** **as Native** **% (***n***)**	**Self-identified** **as Dine** **% (***n***)**
**2018 ISEP (In person)**	11	73 (8)	15–17	100 (11)	91 (10)
**2019 ISEP (In person)**	16	75 (12)	14–17	94 (15)	100 (16)
**2020 ISEP (Virtual)**	10	80 (8)	15–18	100 (10)	100 (10)
**2021 ISEP (Virtual)**	17	82 (12)	14–18	100 (17)	94 (15)

In the four cohorts of ISEP, most students reported that the program influenced their knowledge and awareness of careers in the health field. A majority of students agreed or strongly agreed that the program provided hands-on experience; 82% (9 students) in the 2018 cohort, 100% (16 students) in the 2019 cohort, 80% (8 students) in the 2020 cohort, and 100% (17) in the 2021 virtual cohort (Table 4). Most students felt that the program met their expectations; 73% (8 students) in the 2018 cohort and 100% (16 students) in the 2019 cohort, 100% (10 students) in the 2020 cohort, and 100% (17 students) in the 2021 cohort.

**Table 4:** ISEP acceptability and recommendations.

**Acceptability of the program**	**2018 (11 students)**	**2019 (16 students)**	**2020 (10 students)**	**2021 (17 students)**
**Thoughts of ISEP**	• 91% agree/strongly agree that the program provided hands-on experience in health-related research • 81.8% agree/strongly agree that they were able to learn about and consider careers in the health field • 72% agree/strongly agree ISEP met their expectations	• 100% agree/strongly agree that the program provided hands-on experience in health-related research • 100% agree/strongly agree that they were able to learn about and consider careers in the health field • 100% agree/strongly agree ISEP met their expectations	• 80% agree/strongly agree that the program provided hands-on experience in health-related research • 100% agree/strongly agree that they were able to learn about and consider careers in the health field • 100% agree/strongly agree ISEP met their expectations	• 100% agree/strongly agree that the program provided hands-on experience in health-related research • 100% agree/strongly agree that they were able to learn about and consider careers in the health field • 100% agree/strongly agree ISEP met their expectations
**Recommendations for improvement**	• Add more topics that relate to culture • More time to create digital story • More structure/clear schedule • Teambuilding/games (outside) • Pick own roommates • Clear program expectations/what to bring • More hands-on/problem-solving activities about public health issues	• Increase exercise time, class breaks and free time • More hands-on experiences • Invite more public health guest presenters • More time with SREP students • More time on DST (pre-planning before ISEP to gather photos) • Time management • Add a laptop to supplies needed	• Better hours • More hands-on activities to get people moving out of their seats • More time • Keep involving public health personnel in the program • Seeing each other in person	• Lessons on video editing • More time to work in groups • Have college students work with ISEP • Return to in person learning • Invite college students from diverse backgrounds to the college panel • Include more peer mentors
**Length of ISEP**	• Make program longer	• Make program longer (>1 week)	• Make program longer	• Make program longer (>1 week)

#### Program Acceptability

Students provided feedback about what they liked best about ISEP. All four cohorts of ISEP students consistently expressed that they liked the program staff, fellow students, peer mentors, guest presenters, and opportunities to get to know each other.

“The thing I liked most about the program was learning about all of the people who contribute to the health and wellbeing of my community. I also enjoyed getting up in the morning to work out and meeting amazing people…” (2018 student)

“Learning about how leaders have impacted our community and the instructors have made me feel like I can do the same also in the future. The program educated and informed me about public health, college, and life choices. The information taught has given me experience and knowledge I need for my future, plus the instructors were very helpful, nice, caring, and motivating.” (2018 student)

“I liked how we had interacced [*sic*] in with the SREP (Summer Research Enhancement Program) students, it was an experience to hear their college experiences, to help us out.” (2019 student)

“I liked the staff, the instructor, the peer mentors, the other students best about the program. They all were very helpful and supportive of me throughout the week. All of the staff that presented throughout the days were all so kind and they wanted to see me and the others succeed and be the best that we can be. That made me feel good about the program.” (2019 student)

“I loved the staff and how open everyone was. I liked this because the program can be perfectly planned, but it is the people who make it enjoyable. Furthermore, I liked how everyone learned how to work together and the icebreakers.” (2020 student)

“I liked that everyone involved played an amazing role in this program. I got to know a lot of people and make good connections. I believe these connections will last a lifetime and help me better my life and education in the future.” (2020 student)

“I liked brainstorming with my groups, and discussing our topic. Another thing I like was hearing the different health professional speak, they had so much information to give.” (2021 student)

“I like how we can communicate with each other since it helps with my communication skills.” (2021 student)

Students from the in-person cohorts of 2018 and 2019, expressed that they liked the program structure with hands-on and outdoor activities that take place on a college campus.

“I liked that it was treated somewhat like a college class because it sorta gave a look into a college student's life. I also liked that we didn't just stay indoors all day like a high school class. I also like that we talked about college and what to expect when we get there, it was inspiring to hear stories.” (2018 student)

#### Student Digital Storytelling Videos

In the 2018 cohort, four digital stories were developed by students that focused on health disparities and social determinants of health (SDOH) relevant to the Navajo Nation: homelessness, mental health, environmental health, and alcoholism. In the 2019 cohort, four digital stories were developed by students that focused on public health career paths in the following areas: family and child health, behavioral health, epidemiology, and environmental health. Both cohorts of students developed high quality digital stories using voice overs, pictures and videos. However, in 2019, the students featured two Indigenous public health professionals in their videos. The interviewees discussed their educational background and job duties as an epidemiologist and environmental scientist. In 2020 and 2021 students continued to virtually interview public health professionals to learn more about public health career paths for their digital stories on epidemiology, biostatistics, and global health. Students used break out rooms in zoom to collaborate virtually on these team based digital stories.

#### Cultural Relevance

All four ISEP cohorts strongly agreed or agreed (100%) that they learned how to advocate for the health of their communities. Students gained more cultural insights, one student from 2018 recounts, “I got to understand how important my culture is and how there is so much i [*sic*] can teach with just *Hózhó* and *ke*.” Students also identified as resilient, “[I can] overcome any obstacle no matter the difficulty because I am indigenous and resilient, I can do it.” Students stated appreciation for the Public Health Leadership series that provided access to relatable Native American public health role models and the professional connections created during ISEP.

“I liked how we had visitors who presented information and how they also gave us helpful information about college, being resilient.” (2020 student)

“I like the guest speakers because they wanted to help people in public health and that is exactly what they are doing to achieve that goal and I liked the ice breakers because I got to know everyone and I liked how they all participated.” (2021 student)

“I liked the guest speakers. They were very informative, encouraging, and a great reminder that we as high school ISEP students also can achieve great things.” (2021 student).

#### Program Improvement

Overall many students stated that they did not have any recommendations for improvement because they liked the current program. One common recommendation among all four cohorts was to increase the length of ISEP for longer than 1 week. The 2018 and 2019 ISEP cohorts recommended to incorporate more activities, cultural components, and guest speakers (Table 4). Many of these suggestions were implemented in ISEP 2020 and 2021. Recommendations from the 2020 and 2021 cohorts included more movement activities to get students out of their seats, especially in a virtual environment, increase time for digital stories, include more peer mentors, invite college students to work with high school students, and a return to in-person learning. Another student from ISEP 2020 shared appreciation for the Public Health Leadership series, “I suggest to keep involving great public health personnel in the program. I loved meeting new role models and hearing their stories. I would love to meet more in the future.”

## Discussion

Overall ISEP students expressed appreciation of program staff, fellow students, peer mentors, guest presenters, and the hands-on learning experiences in both in-person and virtual college environments. Our findings are in alignment with other health professional career programs that provide an experiential approach in creating pathways into health professions programs (5, 9, 10, 22). The Navajo NARCH Partnership met its goal of increasing exposure to public health professions and encouraging students to continue on their health professions pathway while still in high school.

### Lessons Learned

Even though the majority of participants agreed/strongly agreed that the program provided hands-on experiences in health-related research, both cohorts requested more hands-on problem solving activities about public health issues. The first cohort recommended the addition of more culturally related topics. Both cohorts requested the incorporation of more teambuilding, longer amount of time dedicated to the digital storytelling process and an overall increase in program duration to span over 1 week in length. While the second cohort suggested integrating more guest speakers and more interaction/contact time with students from the SREP undergraduate program. Kahn et al. stated that resilient teams make for positive student experiences and the ISEP evaluation findings affirm that cohesive teams have a positive impact (11). Students consistently stated how the staff, peer mentors, and guest speakers had a direct positive influence on their learning experiences.

Another valuable lesson resonates with the concept of cultural relevance. The findings report a clear need for thoughtful integration of culturally concordant role models in high school public health pathway programs. The more we can involve community members and connect high school students with Native American public health professionals, specifically role models from their own communities, the more positive impact programs can have on high school students' career pathways.

### Program Strengths/Limitations

ISEP reaches students at an early stage in their career planning. Ultimately the collaborations between high schools and the Navajo NARCH Partnership may serve to build capacity in Diné communities by developing future health professionals to implement culturally congruent public health practices and interventions. While ISEP was highly acceptable to the participants, efforts are needed to increase the length of the summer program to address the recommendations for improvement. The current evaluation data only measures individual cohorts, while this is a weakness of this study, the team plans to follow up with all ISEP cohorts (2018–2021) to determine the longitudinal impact of ISEP. Preliminary steps have been taken to learn about past ISEP participants' career goals, college enrollment, college major, studies or works in a health field, and the peer mentor impact on student success.

Another limitation of these cohorts is the small sample size. Since this program is for high school students, future recruitment needs to focus on reaching not only potential students, but also their parents, guardians, teachers, academic counselors, and school administrators. Expanding recruitment efforts to those who teach and guide students will increase awareness of the program, promote participation and ultimately increase interest in STEM-related health profession careers among high school students.

## Conclusion

ISEP demonstrated programmatic success, by achieving the overall learning objectives. All students increased their knowledge of public health careers and research opportunities on the Navajo Nation. ISEP participants' narratives support the benefits of identifying with successful Native American public health professionals, undergraduate and graduate students. Diné philosophies and beliefs were applied to career pathway planning with high school students and 100% of participants stated that the program met their expectations. An unintended benefit of peer mentoring that came about in the first year of ISEP was when participants of the first cohort requested to attend ISEP again. Due to the demand the ISEP team fostered a peer mentor strategy that provides leadership and communication training as well as leadership opportunities during ISEP. As indicated by the results, the following cohorts of ISEP students appreciated this added level of mentorship by feeling supported by the peer mentors.

While these findings are positive, the value of ISEP will be found as the team explores the impacts of ISEP. The next planned assessment will explore if past ISEP participants select public health or health sciences as a college major and then the team will study the long-term impact of students choosing public health or health sciences careers. The team's next assessment will require a follow-up strategy to be developed and implemented for long term tracking to determine the true impact of ISEP.

## Data Availability Statement

The datasets presented in this article are not readily available because the data belongs to the Navajo Nation, according to the Navajo Research Act and longstanding IRB policy, so any data sharing would have to be specifically approved by them, not by the authors. The datasets are small and include details that could potentially reveal the identity of individual subjects. Requests to access the datasets should be directed to Nicolette I. Teufel-Shone at Nicky.Teufel@nau.edu.

## Ethics Statement

The studies involving human participants were reviewed and approved by Navajo Nation Human Research Review Board. Participants who were 18 years old or older provided their own consent. Anyone under 18 years old provided written informed consent and consent was also provided by the participants' legal guardian/next of kin.

## Author Contributions

All authors contributed to writing, editing, and approving the article for publication.

## Funding

This work was supported by the National Institute on Minority Health and Health Disparities of the National Institutes of Health under Award Number 5S06GM123550.

## Author Disclaimer

The content is solely the responsibility of the authors and does not necessarily represent the official views of the National Institutes of Health.

## Conflict of Interest

The authors declare that the research was conducted in the absence of any commercial or financial relationships that could be construed as a potential conflict of interest.

## Publisher's Note

All claims expressed in this article are solely those of the authors and do not necessarily represent those of their affiliated organizations, or those of the publisher, the editors and the reviewers. Any product that may be evaluated in this article, or claim that may be made by its manufacturer, is not guaranteed or endorsed by the publisher.
